# Distinct Inflammatory Phenotypes Are Associated With Subclinical and Clinical Cardiovascular Disease in People With Human Immunodeficiency Virus

**DOI:** 10.1093/infdis/jiae007

**Published:** 2024-01-12

**Authors:** Padraig McGettrick, Willard Tinago, Julie O’Brien, Sarah Miles, Leo Lawler, Alejandro Garcia-Leon, Niall Mahon, John Lambert, Gerard Sheehan, Alan Landay, Caroline A Sabin, Aoife G Cotter, Patrick W G Mallon, for the HIV Understanding the Pathology of Comorbid Disease in HIV-Infected Individuals With Coronary Artery Disease (UPBEAT) Study Group and the All Ireland Infectious Diseases (AIID) Cohort Study, P McGettrick, E Alvarez Barco, W Tinago, A Garcia-Leon, A McDermott, T McGinty, A G Cotter, A Macken, P W G Mallon, E Kavanagh, G McCarthy, G Sheehan, J Lambert, W Powderly, J Compston, C Sabin, A Cotter, E Muldoon, G Sheehan, T McGinty, J S Lambert, S Green, K Leamy, G Kenny, K McCann, R McCann, C O’Broin, S Waqas, S Savinelli, E Feeney, P W G Mallon, A Garcia Leon, S Miles, D Alalwan, R Negi, E de Barra, S McConkey, K Hurley, I Sulaiman, M Horgan, C Sadlier, J Eustace, C Kelly, T Bracken, B Whelan, J Low, O Yousif, B McNicholas, G Courtney, P Gavin

**Affiliations:** Centre for Experimental Pathogen Host Research, University College Dublin, Dublin, Ireland; Department of Infectious Diseases, Mater Misericordiae University Hospital, Dublin, Ireland; Centre for Experimental Pathogen Host Research, University College Dublin, Dublin, Ireland; Department of Radiology, University Hospital Limerick, Limerick, Ireland; Centre for Experimental Pathogen Host Research, University College Dublin, Dublin, Ireland; Department of Radiology, Mater Misericordiae University Hospital, Dublin, Ireland; Centre for Experimental Pathogen Host Research, University College Dublin, Dublin, Ireland; Department of Cardiology, Mater Misericordiae University Hospital, Dublin, Ireland; Department of Infectious Diseases, Mater Misericordiae University Hospital, Dublin, Ireland; Department of Infectious Diseases, Mater Misericordiae University Hospital, Dublin, Ireland; Department of Internal Medicine, Rush University Medical Center, Chicago, Illinois, United States of America; Institute for Global Health, University College London, London, United Kingdom; Department of Infectious Diseases, Mater Misericordiae University Hospital, Dublin, Ireland; Centre for Experimental Pathogen Host Research, University College Dublin, Dublin, Ireland; Department of Infectious Diseases, St Vincent’s University Hospital, Dublin, Ireland

**Keywords:** CVD, inflammation, subclinical CAD, Inflammatory phenotypes, HIV

## Abstract

Despite inflammation being implicated in cardiovascular disease (CVD) in people with human immunodeficiency virus (PWH), considerable heterogeneity within populations of PWH exists. Stratifying CVD risk based on inflammatory phenotype could play an important role. Using principal component analyses and unsupervised hierarchical clustering, we examined 38 biomarkers to identify inflammatory phenotypes in 2 independent cohorts of PWH. We identified 3 distinct inflammatory clusters present in both cohorts that were associated with altered risk of both subclinical CVD (cohort 1) and prevalent clinical CVD (cohort 2) after adjusting for CVD risk factors. These data support precision medicine approaches to enhance CVD risk assessment in PWH.

Cardiovascular disease (CVD) is a leading cause of death in adults with human immunodeficiency virus (HIV) on antiretroviral therapy (ART) [1] with traditional CVD risk factors, although prevalent in people with HIV (PWH), not fully accounting for the additional observed risk [2].

Multiple inflammatory pathways are implicated in the pathogenesis of atherosclerosis, including endothelial activation, vascular inflammation [3], and type 1 T-helper (Th1) cell activation [4], all contributing to coronary plaque development and instability [5]. Innate immune activation and systemic inflammation are associated with subclinical coronary artery disease (CAD) in PWH [6], with high-sensitivity C-reactive protein (hsCRP) and interleukin (IL) 6 associated with worse outcomes [7].

However, whether 1 specific inflammatory dysfunction predominates in HIV-related CVD is unknown and whether a specific, high-risk inflammatory phenotype exists in PWH remains unclear. To address these data gaps, we aimed to explore inflammatory phenotypes in a cohort of individuals with and without HIV, with no known history of CAD, to determine how inflammatory phenotypes associate with subclinical CAD, measured by coronary computed tomographic angiography (CCTA) and, in a larger independent cohort of PWH, prevalent clinical CVD.

## METHODS

Inflammatory phenotypes were examined in 2 separate cohort studies: Understanding the Pathology of Comorbid Disease in HIV-Infected Individuals With Coronary Artery Disease (HIV UPBEAT CAD) substudy, a cross-sectional study of PWH and HIV-negative subjects (Supplementary Materials); and the All Ireland Infectious Diseases (AIID) Cohort, a prospective, multicenter cohort study enrolling people presenting to the infectious diseases services in a number of clinical sites in Ireland, with only PWH on suppressive ART included in this analysis. Within the HIV UPBEAT CAD substudy, PWH and controls were considered for inclusion if they were >40 years old with no known history of CVD and were propensity score matched for traditional CAD risk factors, ensuring an even distribution across both groups. Participants underwent CCTA to assess for subclinical CAD, reported as presence of any plaque, noncalcified plaque, partially calcified plaque, and calcified plaque, in addition to 2 coronary calcification scores: the Agatston score and calcium volume score (Supplementary Materials).

Participants of both studies attended for fasting blood sample collection and CVD risk assessment, with data collected on prevalence of comorbidities including CVD, hypertension, dyslipidemia, and chronic kidney disease (CKD) at time of sample collection.

### Inflammatory Biomarker Analysis

Stored plasma and peripheral blood mononuclear cells were used to test for 28 inflammatory biomarkers (reflecting systemic inflammation, innate immune activation, Th1 response, microbial translocation, gut intestinal barrier function, endothelial inflammation, coagulation, and T-cell differentiation and modulation) using custom multiplex chemiluminescence immunoassays on Luminex (R&D Systems, St Paul, Minnesota) and Meso Scale Discovery (Rockland, Maryland) platforms (Supplementary Table 1) and 10 T-cell surface biomarkers using flow cytometry (Beckman Coulter Cytoflex) and analyzed using FlowJo version 10 (BD Biosciences) software.

### Statistical Analysis

All biomarker data were entered into a principal component analysis (PCA) followed by unsupervised hierarchical clustering on the principal components to identify biomarker-derived clusters (Supplementary Materials). Individual biomarkers’ contribution to cluster formation was determined using R package FactoMineR with influencing variables sorted from the most to the less influential.

We used logistic regression to investigate relationships between derived clusters and endpoints of interest: in the UPBEAT CAD study, subclinical CAD as measured by CCTA with calcification scores both reported as categorical variables (Agatston score >100, calcium volume score >100); in the AIID Cohort study, prevalent vascular disease (history of CVD, hypertension or CKD as previously described [8]) and CVD (myocardial infarction, angina, percutaneous coronary intervention, stroke, transient ischemic attack, and/or peripheral vascular disease). Clinical and demographic variables associated (*P* < .1) with endpoints on univariate analysis were included in multivariable models. Analysis was undertaken using Stata 13.1 (StataCorp, College Station, Texas) and R V3.5.1 (R Foundation for Statistical Computing, Vienna, Austria) software.

## RESULTS

Of 100 HIV UPBEAT CAD participants (50% PWH), median age was 50 (interquartile range [IQR], 46–56) years, 73.3% were male, 76.2% White, and 22% were current smokers, with CVD risk factors evenly distributed between PWH and controls through propensity matching. However some differences remained; PWH were more likely to be on statins and have lower high-density lipoprotein cholesterol and diastolic blood pressure (Supplementary Table 2).

Following PCA and unsupervised hierarchical clustering analysis (HCA), 3 distinct inflammatory clusters were identified: cluster 1 comprising 41% (32.5% PWH) of participants, cluster 2 comprising 40% (72.5% PWH), and cluster 3 comprising 19% (50% PWH).

Cluster 1 (reference cluster) was characterized by lower inflammation (Supplementary Figure 1), spanning both innate immunity (lower tumor necrosis factor [TNF]–α, TNF receptors 1 and 2, and IL-1RA) and T-cell immunity (lower CD4^+^ and CD8^+^ T-cell senescence and activation). Cluster 2 (innate immune/inflamed) comprised a significantly higher proportion of PWH (72.5%), had elevated biomarkers of innate immune activation (soluble CD163 [sCD163], monocyte chemoattractant protein-1 [MCP-1]), coagulation (D-dimer, soluble CD40L), endothelial function (soluble intracellular adhesion molecule 1 [sICAM-1], vascular cell adhesion molecule 1 [VCAM-1], E-selectin, and P-selectin), microbial translocation (sCD14 and lipopolysaccararide binding protein [LBP]), and T-cell senescence, markers all implicated in prior inflammatory studies in PWH [9]. In contrast, cluster 3 (gut/T-cell/inflamed) comprised 50% PWH who had elevated biomarkers of gut epithelial dysfunction (Intestinal fatty acid binding protein [I-FABP]), T-cell differentiation and regulation (IL-2, IL-4, IL-12, IL-10, and interferon gamma [IFN-γ]), and systemic inflammation (TNF, IL-6, and IL-1β).

Despite these different inflammatory phenotypes, most demographic characteristics were similar across the 3 clusters (Table 1), with the exception of HIV status, which was overrepresented in cluster 2 (innate immune/inflamed).

**Table 1.** Baseline Demographics and Coronary Artery Disease (CAD) Risk Factors of Participants of the UPBEAT CAD Study and All Ireland Infectious Diseases Cohort Study, Stratified by Inflammatory Cluster

Characteristic	HIV UPBEAT CAD Cohort (n = 100)				AIID Cohort (n = 277)			
	Cluster 1	Cluster 2	Cluster 3	*P* Value	Cluster 1	Cluster 2	Cluster 3	*P* Value
Number of participants (%)	41 (41%)	40 (40%)	19 (19%)		148 (53.4%)	100 (36.1%)	29 (10.5%)	
Age, y	48 (45–52)	51 (46–61)	49 (46–55)	.09	43 (38–50)	45 (40–50)	45 (40–48)	.59
Male	27 (67.5%)	31 (77.5%)	13 (72.2%)	.61	81 (54.7%)	59 (59.0%)	19 (65.5%)	.52
White	26 (65.0%)	34 (85.0%)	14 (77.8%)	.11	64 (43.2%)	48 (48.0%)	15 (51.7%)	.91
Current smoker	13 (34.2%)	6 (15.4%)	2 (11.8%)	.07	30 (23.8%)	18 (23.7%)	9 (36.0%)	.28
Living with HIV	13 (32.5%)	29 (72.5%)	9 (50.0%)	.002	148 (100%)	100 (100%)	29 (100%)	
History of IVDU	3 (7.5%)	4 (10%)	1 (5.6%)	.83	15 (10.1%)	15 (15.0%)	2 (6.9%)	.54
Diabetes	2 (4.9%)	2 (5.1%)	0 (0)	.62	7 (4.8%)	4 (4%)	3 (10.3%)	.38
On statins	8 (19.5%)	15 (38.5%)	6 (33.3%)	.17	29 (23.2%)	21 (27.6%)	6 (25%)	.78
BMI, kg/m^2^	26.5 (24.4–30.7)	27.8 (24.1–30.6)	29.6 (27.0–33.1)	.19	27 (23–30)	26 (23–29)	25.5 (21.5–31)	.75
Systolic BP, mm Hg	133 (122–145)	133 (127143)	141 (135–156)	.21	125 (114–136)	126 (113–140)	130 (120–140)	.56
Diastolic BP, mm Hg	82 (75–90)	82 (77–89)	87 (83–92)	.21	74 (65–81)	76 (67–81)	75 (67–82)	.44
Total cholesterol, mmol/L	5.25 (4.65–6.1)	4.6 (4.0–5.3)	5.0 (4.2–5.5)	.04	4.9 (4.3–5.7)	4.9 (4.05–5.5)	4.3 (3.9–5.2)	.22
LDL cholesterol, mmol/L	3.3 (2.6–4.10)	2.8 (2.2–3.5)	3.0 (2.4–3.8)	.08	3.1 (2.6–3.7)	3.1 (2.3–3.6)	2.6 (2.4–3.3)	.12
HDL cholesterol, mmol/L	1.42 (1.13–1.64)	1.26 (1.01–1.38)	1.25 (.94–1.59)	.02	1.21 (.99–1.49)	1.2 (1.04–1.44)	1.27 (.96–1.48)	.79
HIV-related immunological indices	n = 13	n = 29	n = 9		n = 148	n = 100	n = 29	
Current CD4 T-cell count, cells/μL	688 (512–1009)	706 (516–893)	710 (521–957)	.99	681 (512–885)	640 (450–909)	717 (619–872)	.45
Current CD4 CD8 T-cell ratio	0.95 (.73–1.04)	0.86 (.69–1.30)	0.86 (.72–1.53)	.92	0.84 (.58–1.15)	0.83 (.54–1.14)	1.07 (.73–1.29)	.15
Nadir CD4^+^ T-cell count, cells/μL	224 (158–292)	252 (94–345)	139 (79–333)	.757	214 (103–386)	237 (111–394)	322 (205–439)	.19
ART treatment duration, y	10.6 (7.24–14.44)	10.37 (7.36–15.40)	8.65 (7.29–10.26)	.430	4.64 (2.02–10.96)	4.73 (2.04–9.11)	4.75 (1.87–10.46)	.76
Primary ART classes								
INSTI	4 (30.8%)	16 (57.1%)	4 (50.0%)	.91	79 (53.4%)	50 (50.5%)	16 (55.2%)	.83
NNRTI	5 (38.5%)	10 (35.7%)	3 (37.5%)		37 (25%)	30 (30.3%)	6 (20.7%)	
PI	4 (30.8%)	2 (7.1%)	1 (12.5%)		32 (21.6%)	19 (19.2%)	7 (24.1%)	

Data are presented as No. (%) or median (interquartile range).

Between-group differences were assessed using Kruskal–Wallis and χ^2^ testing.

Abbreviations: AIID, All Ireland Infectious Diseases; ART, antiretroviral therapy; BMI, body mass index; BP, blood pressure; CAD, coronary artery disease; HDL, high-density lipoprotein; HIV, human immunodeficiency virus; INSTI, integrase strand transfer inhibitor; IVDU, intravenous drug use; LDL, low-density lipoprotein; NNRTI, nonnucleoside reverse transcriptase inhibitor; PI, protease inhibitor; UPBEAT CAD, Understanding the Pathology of Comorbid Disease in HIV-Infected Individuals With Coronary Artery Disease.

Overall, 36% (n = 36) of the cohort had subclinical CAD on CCTA; 32% (n = 32) calcified plaque, 20% (n = 20) partially calcified plaque, and 11% (n = 11) noncalcified plaque. Nine percent (n = 9) had a moderate-to-severe stenosis (>50% luminal stenosis). Sixteen percent had Agatston score >100 units and 11% had calcium volume score >100. Coronary plaque burden was similar between PWH and controls, although more controls had coronary calcification (Supplementary Table 3).

**Figure 1.**
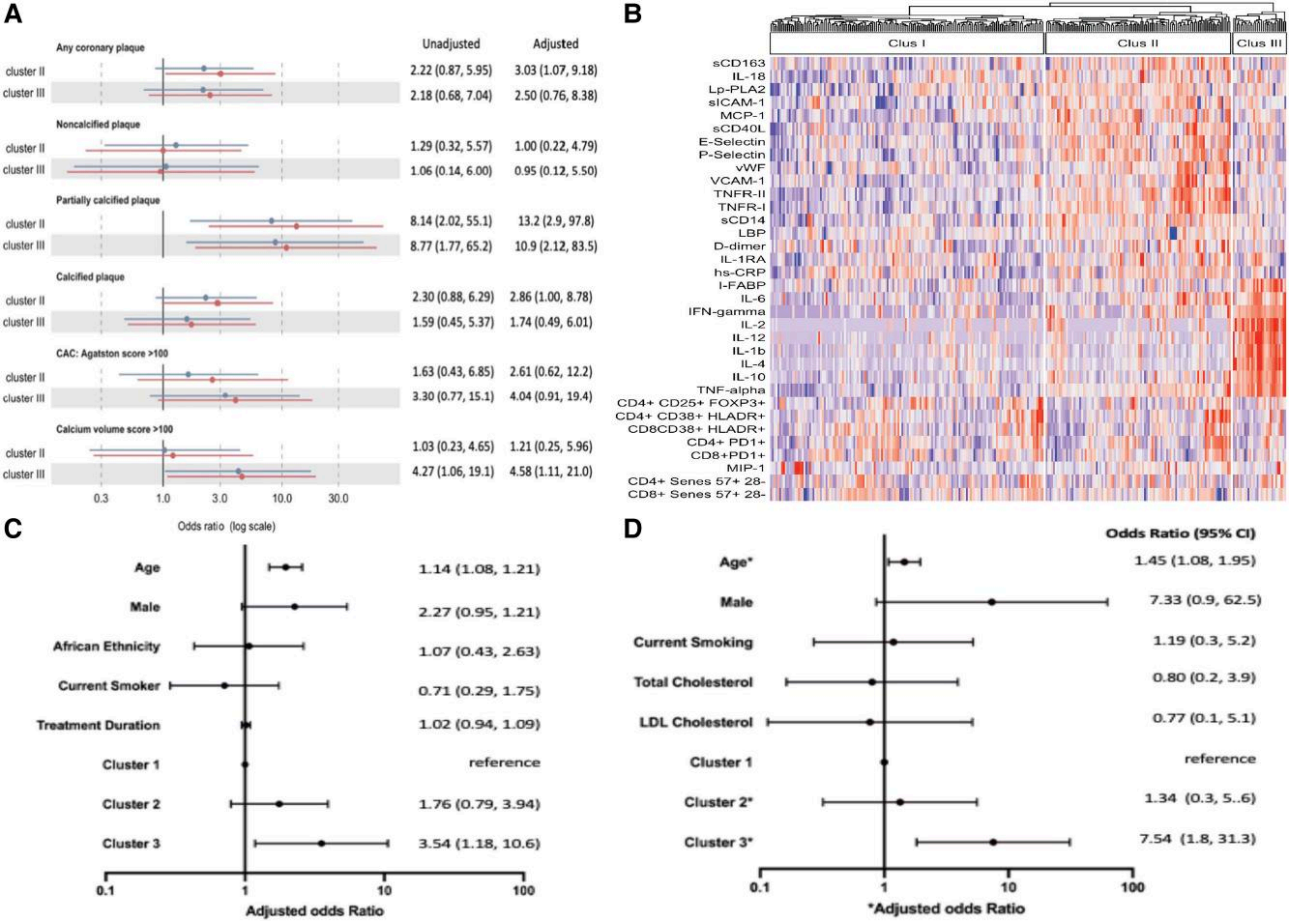
*A*, Associations between inflammatory phenotypes (Supplementary Figure 1) and subclinical coronary artery disease (CAD) in unadjusted and adjusted (for HIV status) analyses in the HIV UPBEAT CAD substudy. *B*, Biomarker contribution to cluster formation in the All Ireland Infectious Diseases (AIID) Cohort and *C*, associations with prevalent vascular disease adjusted for age, male gender, ethnicity, antiretroviral therapy duration, and smoking and *D*, prevalent cardiovascular disease. *adjusted for age and gender. Abbreviations: CAC, coronary artery calcification; CI, confidence interval; LDL, low-density lipoprotein.

In analyses adjusted for HIV status, cluster 2 was significantly associated with presence of any coronary plaque (odds ratio [OR], 3.0 [95% confidence interval {CI}, 1.1–9.2]), partially calcified plaque (OR, 13.2 [95% CI, 2.9–97.8]), and calcified plaque (OR, 2.9 [95% CI, 1.0–8.8]) relative to cluster 1 (reference cluster). In contrast, cluster 3 (gut/T-cell/inflamed) was associated only with partially calcified plaque (OR, 10.9 [95% CI, 2.1–83.5]) but, unlike cluster 2, was also associated with greater coronary calcification by calcification scores (OR, 4.3 [95% CI, 1.1–19.1]) compared to cluster 1 (Figure 1*A*), with further adjustment for age, smoking status, and statin use strengthening these associations (Agatston score >100: OR, 11.8 [95% CI, .9–150]; calcium volume score >100: OR, 15.5 [95% CI, .94–256]).

We next examined inflammatory phenotypes in the larger AIID cohort. Of 277 PWH, median age was 44 (IQR, 39–50) years, 57.4% were male, 45.8% White, and 43.0% African. The median time since HIV diagnosis and ART commencement was 10 (IQR, 5–16) years and 5 (IQR, 2–11) years, respectively, with median current CD4^+^ T-cell count of 651 (IQR, 460–833) cells/μL.

Following inflammatory biomarker PCA and HCA, 3 distinct inflammatory clusters were identified: cluster 1 comprising 148 (53.4%) individuals, cluster 2 comprising 100 (36.1%) individuals, and cluster 3 comprising 29 (10.5%) individuals. The demographics and HIV-related characteristics were similar across the 3 clusters (Table 1). We found almost identical patterns of inflammation contributing to the 3 clusters, with the exception of T-cell senescent markers, which no longer discriminated between clusters in this cohort of exclusively PWH (Figure 1*B*). Cluster 1 was again characterized by overall lower inflammation while clusters 2 and 3 were characterized by high inflammatory markers: cluster 2 by higher markers of gut microbial translocation (sCD14, LBP), innate immune activation (sCD163, MCP-1), systemic inflammation (TNF receptors 1 and 2, hsCRP), and vascular endothelial function (P-selectin, E-selectin, sICAM-1, sVCAM-1), and cluster 3 by gut epithelial dysfunction (I-FABP), T-cell differentiation and regulation (IFN-γ, IL-2, IL-4, IL-12, IL-10), and systemic inflammation (TNF-α, IL-6, IL-1β).

When comparing inflammatory profiles to prevalent vascular comorbidities (n = 75) and CVD (n = 15), individuals in cluster 3 (gut/T-cell/inflamed) were more than twice as likely to have vascular comorbidities relative to those in cluster 1 (reference) (OR, 2.26 [95% CI, .95–5.38]; *P* = .06), an association that persisted in fully adjusted analyses (Figure 1*C*) and in sensitivity analysis adjusting for other cardiovascular risk factors (history of diabetes mellitus, hypertension, and dyslipidemia) (OR, 8.03 [95% CI, .76–84.5]). Similarly, cluster 3 (gut/immune activated) was also significantly associated with prevalent CVD (OR, 7.24 [95% CI, 1.8–28.9]), an association which persisted on adjustment for other significantly associated variables (Figure 1*D*) and in fully adjusted analyses (OR, 7.06 [95% CI, 1.16–42.8]; *P* = .03).

## DISCUSSION

In this study, we identified clusters of individuals with HIV with distinct inflammatory phenotypes in 2 independent cohorts, which not only shared similar inflammatory characteristics but were also associated with both subclinical CAD and prevalent CVD. Importantly, that similar inflammatory profiles were observed across 2 separate cohorts, and were independently associated with different CVD measures (clinical and subclinical), with associations persisting after correction for relevant clinical factors, strongly suggests a causal link between the observed inflammatory patterns and CVD.

The pattern of inflammation observed in cluster 2 within both cohorts is typical of that described in PWH [3], with elevated markers of microbial translocation (sCD14, LBP), innate immune activation (sCD163, MCP-1), and vascular inflammation (VCAM, sICAM, P-selectin, and E-selectin). This is reflected by the higher prevalence of PWH in cluster 2 in the UPBEAT CAD substudy analysis. That we observed a similar inflammatory phenotype in the AIID Cohort analysis, which consisted exclusively of PWH, suggests that this pattern of inflammation persists as a differentiating phenotype even within the treated PWH population. That individuals with this phenotype had higher prevalence of coronary plaque and partially calcified plaque on CCTA suggests a benefit in identifying individuals displaying a similar inflammatory phenotype for targeted primary prevention strategies.

In contrast, cluster 3 displayed higher markers of gut epithelial dysfunction, T-cell stimulation, and systemic inflammation within both cohorts and was distinguished from cluster 2 in the HIV UPBEAT CAD study by being significantly associated with higher coronary artery calcification and partially calcified plaque. The higher I-FABP in this cluster likely reflects increased enterocyte turnover due to systemic metabolic derangements rather than, in the absence of suggestive markers (sCD14, LBP), gut microbial translocation. Interestingly, it was this inflammatory phenotype in the AIID Cohort study, comprising exclusively PWH, that was most associated with prevalent vascular and CVD. That this phenotype comprised similar proportions of PWH and controls in the UPBEAT CAD study represents an unusual pattern of inflammation that lacks the usual markers of microbial translocation and innate immune activation typical of PWH, and that it was most strongly associated with CVD argues for a causal relationship between this distinct pattern of inflammation and clinical CVD and warrants further research as a potential, novel atherogenic inflammatory phenotype that may involve distinct pathways other than those more commonly seen in HIV infection.

Previous studies have reported inflammatory phenotypes in cohorts of treated PWH similar to those identified in our analysis [10]; however none have been compared to subclinical and clinical CVD. In an analysis of the Pharmacokinetic and Clinical Observations in People Over Fifty (POPPY) cohort, the systemically inflamed/gut epithelial dysfunction grouping was associated with higher estimated CVD risk, again supporting our findings highlighting this phenotype as a particularly high-risk group [11].

Our study has limitations. Although independent cohorts, both analyses were cross-sectional, limiting causality determination and assessment of associations over time. The relatively low prevalence of CVD reduced precision of association estimates despite incorporating propensity score matching to reduce the need for extensive adjustments. Biomarker analysis was conducted on cryopreserved rather than fresh samples; however, quality assurance and control measures were strictly observed to minimize cell death and protein degradation. Finally, although we explored the impact of variables on inflammatory cluster composition, there remains the possibility of unmeasured confounding.

In conclusion, this study has identified clinically relevant inflammatory phenotypes in 2 independent cohorts of people with HIV that are associated with both subclinical CAD and prevalent CVD events. These results provide valuable insights into distinct inflammatory pathways contributing to pathogenesis of CAD in people with HIV and provide evidence for a precision medicine–based approach to enhance CVD risk prediction and future CVD preventive strategies.

## Supplementary Data

Supplementary materials are available at *The Journal of Infectious Diseases* online (http://jid.oxfordjournals.org/). Supplementary materials consist of data provided by the author that are published to benefit the reader. The posted materials are not copyedited. The contents of all supplementary data are the sole responsibility of the authors. Questions or messages regarding errors should be addressed to the author.

## Supplementary Material

jiae007_Supplementary_Data

## Notes

***Acknowledgments*.** The authors thank the UPBEAT CAD substudy and All Ireland Infectious Diseases Cohort study participants and their families for their involvement in this study.

***HIV UPBEAT Study Group.*** Centre for Experimental Pathogen Host Research, University College Dublin, Ireland: P. McGettrick, E. Alvarez Barco, W. Tinago, A. Garcia-Leon, A. McDermott, T. McGinty, A. G. Cotter, A. Macken, P. W. G. Mallon. Mater Misericordiae University Hospital: E. Kavanagh, G. McCarthy, G. Sheehan, J. Lambert. Institute for Public Health, Washington University School of Medicine, St Louis, Missouri: W. Powderly. Department of Medicine, Cambridge Biomedical Campus, Cambridge, UK: J. Compston. Institute for Global Health, University College London, UK: C. Sabin.

***All-Ireland Infectious Diseases Cohort Study Group.*** Mater Misericordiae University Hospital: A. Cotter, E. Muldoon, G. Sheehan, T. McGinty, J. S. Lambert, S. Green, K. Leamy. St Vincent's University Hospital: G. Kenny, K. McCann, R. McCann, C. O’Broin, S. Waqas, S. Savinelli, E. Feeney, P. W. G. Mallon. Centre for Experimental Pathogen Host Research: A. Garcia Leon, S. Miles, D. Alalwan, R. Negi. Beaumont Hospital: E. de Barra, S. McConkey, K. Hurley, I. Sulaiman. University College Cork: M. Horgan, C. Sadlier, J. Eustace. University College Dublin: C. Kelly, T. Bracken. Sligo University Hospital: B. Whelan. Our Lady of Lourdes Hospital: J. Low. Wexford General Hospital: O. Yousif. University Hospital Galway: B. McNicholas. St Luke's Hospital Kilkenny: G. Courtney. Children's Health Ireland: P. Gavin.

***Author contributions.*** All authors contributed substantially to the development of this study and contributed significantly and equally to this manuscript.

***Disclaimer.*** The funders had no role in the study design, data collection and analysis, decision to publish, or preparation of the manuscript.

***Financial support*.** The HIV UPBEAT Cohort Study has received funding from the Health Research Board (HRB) (award number HRA_POR/2010/66; both grants awarded to P. W. G. M. as principal investigator). Funding to pay the Open Access publication charges for this article was provided by University College Dublin through the 82723451 Irish Institutions (IREL affiliated) - Hybrid Open Access Agreement.
