# Supplementary material for: Distinct Inflammatory Phenotypes Are Associated With Subclinical and Clinical Cardiovascular Disease in People With Human Immunodeficiency Virus
Source: J Infect Dis. 2024 Jan 12;230(3):e616–21. doi: 10.1093/infdis/jiae007 (PMC11420771; doi:10.1093/infdis/jiae007)
Supplement: jiae007_Supplementary_Data [file jiae007_supplementary_data.docx]

**Supplementary materials**

HIV UPBEAT CAD Substudy recruitment strategy and procedures

Participants in the HIV UPBEAT CAD substudy were enrolled from within the HIV UPBEAT cohort, a prospective observational study of PWH and uninfected controls. In order to demographically reflect the population of PWH, the control group were recruited from patient family groups, local community and businesses (1). Participants were considered for inclusion in the substudy if they were over 40 years old, had no known history of cardiovascular disease and had attended the most recent follow up visit. People living with HIV were required to be on ART and be virally suppressed in the preceding 9 months. 100 eligible candidates were recruited to the substudy based on propensity score matching incorporating traditional CAD risk factors in addition to HIV status; ensuring an even distribution of CAD risk factors in both PWH and the uninfected control group.

Substudy participants underwent coronary computer tomographic angiography to assess for subclinical CAD, at a single site using a standardised scanning protocol and analysed in a central core lab using Syngo Via reconstruction software (Siemens, Erlangen, Germany). All scans were reported for presence of any plaque, non-calcified plaque, partially calcified and calcified plaque in addition to two coronary calcification scores: the Agatston coronary artery calcification score and calcium volume score by a single study radiologist who was blinded to participants HIV status.

Statistical Analysis: Principal Components Analysis and Hierarchical clustering

Inflammatory biomarker data were entered into a principal component analysis (PCA) to reduce the dimensionality and eliminate redundancy across the dataset with biomarkers log transformed (for approximate normality) and scaled to ensure biomarkers with larger intrinsic variation did not dominate the subsequent PCA. Unsupervised hierarchical clustering was then performed on the principal components (PCs), using Ward’s minimum variance method and squared Euclidean distance as the distance measure, and determining the optimal number of clusters using the Silhouette method. For PCA, we used a single imputation for absolute values of missing biomarker data (ranging from 0.5% to 20%) using the SVDImpute algorithm within the R impute package.

Individual biomarkers contribution in the construction of a cluster was determined using the standardized difference between the cluster conditional mean and the mean of the variable in the cluster (2)*,*using R package *FactoMineR* with influencing variables sorted from the most to the less influential based on the value of their standardized difference (3)

Supplementary Bibliography

1. Cotter AG, Sabin CA, Simelane S, Macken A, Kavanagh E, Brady JJ, et al. Relative contribution of HIV infection, demographics and body mass index to bone mineral density. AIDS. 2014 Sep 10;28(14):2051–60.

2. Lebart L, Morineau A, Piron M. Statistique exploratoire multidimensionnelle. Paris: Dunod; 1995.

3. Lê S, Josse J, Husson F. FactoMineR: An R Package for Multivariate Analysis. Journal of Statistical Software. 2008 Mar 18;25:1–18.

Supplementary Tables and Figures

| Inflammatory Protein Biomarker panel | | |
| --- | --- | --- |
| Systemic Inflammation | | Th1 cytokines |
| hsCRP  IL6  IL1b  IL1RA | TNF  TNFR1  TNFR2 | IL2  IL12  IL18  IFN |
| Innate Immune Activation | | Microbial translocation |
| sCD163  MCP1 | MIP1a | sCD14  LBP |
| Endothelial Inflammation | | T cell modulation |
| s-ICAM  s-VCAM  soluble E selectin  vWF | | IL4  IL10  TSLP |
| Coagulation | | Gut epithelial barrier disruption |
| soluble P selectin  sCD40L | D Dimer | I-FABP |
| T cell immunophenotyping | | |
| Phenotype | | Surface markers |
| T cell (CD4+/CD8+) activation | | CD3+ (CD4+/CD8+) CD38+ HLADR+; |
| T cell (CD4+/CD8+) exhaustion | | CD3+(CD4+/CD8+) PD-1+ |
| T cell (CD4+/CD8+) senescence | | CD3+ (CD4+/CD8+) CD 28- CD57+ |
| terminally differentiated cells | | CD3+ (CD4+/CD8+) CD57+ |
| regulatory T cells | | CD3+ CD4+ CD25+ FoxP3+ |
|  | | |
| hsCRP, high sensitivitiy C Reactive protein; IL, Interleukin; IL1b, interleukin 1 beta; IL1RA, IL-1receptor antagonist; TNFR, tumour necrosis factor receptor; sCD163, soluble cluster of differentiation 163; MCP-1 Monocyte chemoattractant protein; MIP-1, macrophage inflammatory protein; s-ICAM, soluble intercellular adhesion molecule; vwf, von willibrand factor; VCAM1, Vascular cell adhesion molecule; sCD40L, soluble CD40 ligand; IFN, interferon; LBP, LPS binding protein; TSLP, Thymic Stromal Lympoprotein; I-FABP,intestinal fatty acid binding protein; | | |

**Table 1: Protein inflammatory Biomarker and T cell immunophenotyping panel for use in Principal Component Analysis**

|  | | HIV UPBEAT CAD Cohort | | AIID Cohort |
| --- | --- | --- | --- | --- |
|  | | People with HIV | People without HIV |  |
| N | | 50 | 50 | 277 |
| Age (years), median (IQR) | | 49 (45, 59) | 50 (46, 55) | 44 (39, 50 ) |
| Male | | 38 (74.5%) | 34 (70.8%) | 159 (57.4%) |
| Caucasian | | 38 (74.5%) | 37 (77.1%) | 127 (45.8%) |
| Current Smoker | | 11 (21.6%) | 11 (22.9%) | 57 (25.1%) |
| History of Diabetes | | 3 (5.8%) | 1 (2.0%) | 14 (5.1%) |
| Currently using statins | | 24 (49.0%) | 6 (12.5%) | 56 (20.2%) |
| BMI (kg/m^2^), median (IQR) | | 29.1 (24.4, 32.1) | 27.5 (25.0, 29.6) | 26.5 (23, 30) |
| Systolic BP, mm Hg | | 134 (125, 143) | 135 (127, 154) | 126 (114, 137) |
| Diastolic BP, mm Hg | | 82 (74, 88) | 84 (80, 91) | 77 (68, 88) |
|  | |  |  |  |
| Total Cholesterol -mmol/l | | 4.9 (4.3, 5.8) | 5.1 (4.2, 5.7) | 4.9 (4.2, 5.6) |
| LDL cholesterol -mmol/l | | 3.0 (2.5, 3.6) | 3.0 (2.4, 3.75) | 3.0 (2.5, 3.6) |
| HDL cholesterol -mmol/l | | 1.3 (1.07, 1.34) | 1.4 (1.1, 1.7) | 1.2 (1.0, 1.5) |
|  | |  | |  |
| Current CD4+ count (cells/mm^3^), median (IQR) | | 702 (514, 903) | - | 651 (460, 833) |
| Nadir CD4+ count (cells/mm^3^), median (IQR) | | 225 (108, 335) | - | 224 (109, 395) |
| Duration of ART (years), median (IQR) | | 10 (7, 14) | - | 5 (2, 11) |
| Current ART regimen | |  |  |  |
|  | Abacavir | 2 (4%) | - | 50 (18%) |
|  | INSTI | 24(48%) | - | 160 (58%) |
|  | NNRTI | 17 (34%) | - | 78 (28%) |
|  | PI | 7 (14%) | - | 58 (21%) |
| HIV RNA <40 copies/ml | | 48 (94.1%) | - | 277 (100%) |
| IQR; interquartile range; BMI: body mass index; ART: antiretroviral therapy; BP: blood pressure; INSTI: Integrase Strand Transfer Inhibitor; NNRTI: Non-nucleoside reverse transcriptase inhibitor; PI: Protease Inhibitor. | | | | |

**Table 2: Baseline characteristics of the HIV UPBEAT CAD substudy and AIID Cohort study analysis**

|  | | | |
| --- | --- | --- | --- |
|  | People with HIV | HIV uninfected | P Value |
| Any Plaque n(%) | 16 (32.0%) | 19 (38.8%) | 0.48 |
| Non Calcified Plaque n(%) | 7 (14.0%) | 3 (6.1%) | 0.19 |
| Partially calcified Plaque n(%) | 9 (18.0%) | 11 (22.4%) | 0.58 |
| Calcified Plaque n(%) | 15 (30.0%) | 17 (34.7%) | 0.62 |
| Coronary calcification scores | | | |
| Agatston Score *, median (IQR) | 0.0 (0.0, 6.4) | 0.0 (0.0, 66.8) | 0.18 |
| Calcium Volume Score*, Median (IQR) | 0.0 (0.0, 4.9) | 0.0 (0.0, 41.6) | 0.24 |
| Agatston^†^ >100^^^, n(%) | 4 (7.8%) | 12 (24.5%) | 0.02 |
| Calcium Volume Score^^^ >100, n(%) | 2 (3.9%) | 9 (18.4%) | 0.02 |
| Worst Stenosis |  |  | 0.23 |
| No Stenosis | 34 (68%) | 30 (61.2%) |  |
| 1- 49% | 12 (24%) | 14 (28%) |  |
| >50% | 4 (8%) | 5 (10%) |  |
| Calcification scores have been reported as continuous variable(*) and as a binary variable (†) of score greater than or less than 100 units. | | | |

**Table 3: Subclinical CAD as measured by CCTA in HIV UPBEAT CAD Substudy participants**


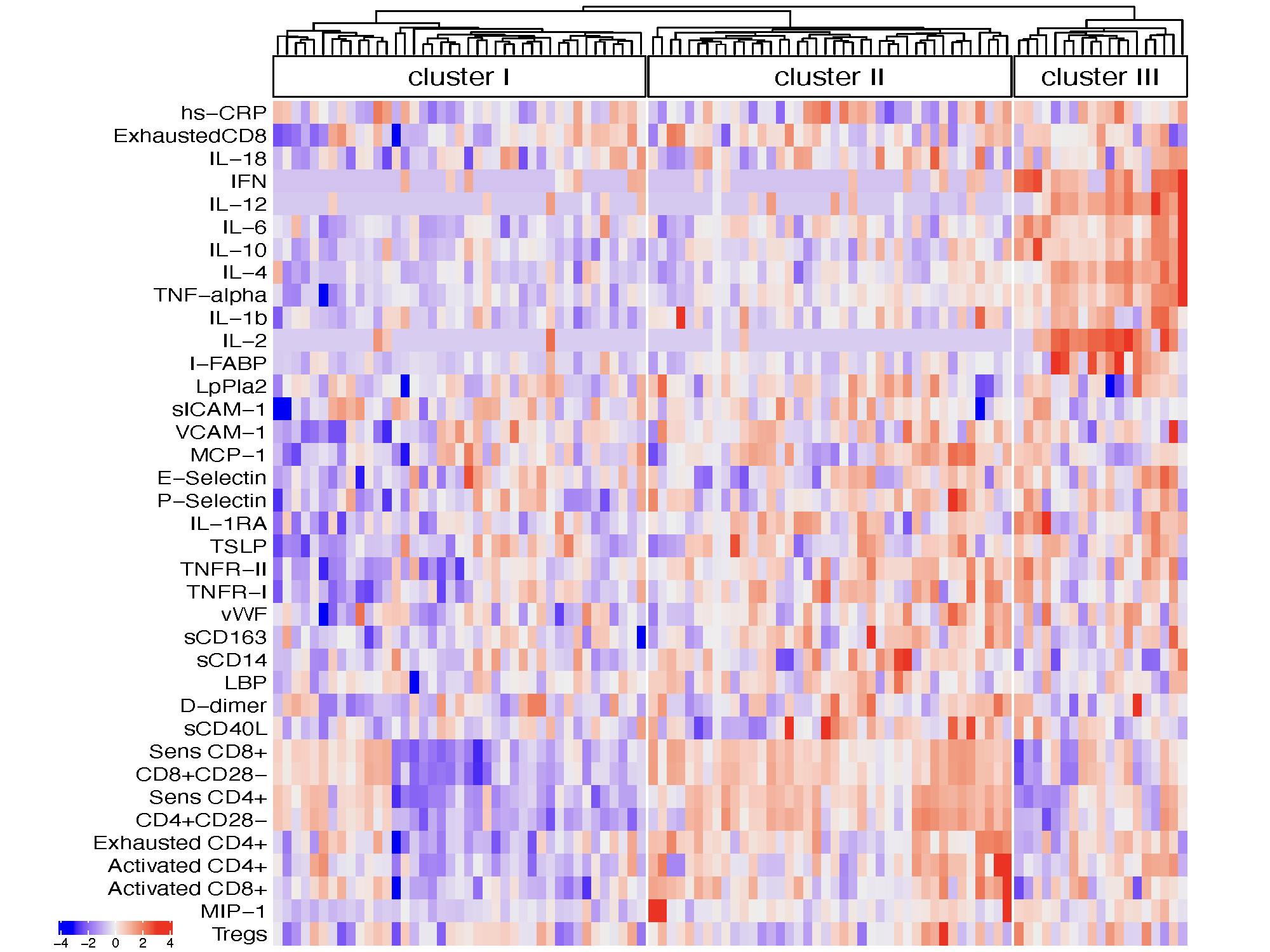


**Figure 1: Heatmap displaying biomarker contribution to Inflammatory phenotype formation in UPBEAT CAD Substudy**

^IL, Interleukin;MCP-1 Monocyte chemoattractant protein; vwf, von willibrand factor; VCAM1, Vascular cell adhesion molecule; TNFR, tumour necrosis factor receptor; LBP, LPS binding protein; IL-1RA, IL-1receptor antagonist; I-FABP,intestinal fatty acid binding protein; MIP-1, macrophage inflammatory protein^
